# Statement of Retraction: Methyltransferase-like 3 silenced inhibited the ferroptosis development via regulating the glutathione peroxidase 4 levels in the intracerebral hemorrhage progression

**DOI:** 10.1080/21655979.2026.2667609

**Published:** 2026-05-21

We, the Editors and Publisher of the journal *Bioengineered*, have retracted the following article from publication.

Zhang, L., Wang, X., Che, W., Yi, Y., Zhou, S., & Feng, Y. (2022). Methyltransferase-like 3 silenced inhibited the ferroptosis development via regulating the glutathione peroxidase 4 levels in the intracerebral hemorrhage progression. *Bioengineered*, 13(6), 14215–14226. https://doi.org/10.1080/21655979.2022.2084494

Since publication, significant concerns have been raised by a third party about the integrity of the data and reported results in the article. Specifically, concerns were raised about alleged image duplication with similar images from an unrelated article:

• Figure 3a, first panel appears to be duplicated with a similar image in Figure 2c from Kang et al, 2022 (https://doi.org/10.1080/21655979.2023.2180591)

• Figure 6a, first panel appears to be duplicated with a similar image in Figure 5c from Kang et al, 2022 (https://doi.org/10.1080/21655979.2023.2180591)

When approached for an explanation, the authors have not responded to our queries and so these serious concerns remain unaddressed.

As verifying the validity of published work is core to the integrity of the scholarly record, we are therefore retracting the article. The authors have been informed of this decision.

We have been informed in our decision-making by our policy on publishing ethics and integrity and COPE guidelines.

The retracted article will remain online to maintain the scholarly record and will be digitally watermarked on each page as ‘Retracted’.

